# Mouse Mammary Tumor Virus Signal Peptide Uses a Novel p97-Dependent and Derlin-Independent Retrotranslocation Mechanism To Escape Proteasomal Degradation

**DOI:** 10.1128/mBio.00328-17

**Published:** 2017-03-28

**Authors:** Hyewon Byun, Poulami Das, Houqing Yu, Alejandro Aleman, Mary M. Lozano, Andreas Matouschek, Jaquelin P. Dudley

**Affiliations:** a Department of Molecular Biosciences, The University of Texas at Austin, Austin, Texas, USA; b Center for Infectious Disease, The University of Texas at Austin, Austin, Texas, USA; c Institute for Cellular and Molecular Biology, The University of Texas at Austin, Austin, Texas, USA; Columbia University

**Keywords:** ERAD, retrotranslocation, retrovirus, signal peptide

## Abstract

Multiple pathogens, including viruses and bacteria, manipulate endoplasmic reticulum-associated degradation (ERAD) to avoid the host immune response and promote their replication. The betaretrovirus mouse mammary tumor virus (MMTV) encodes Rem, which is a precursor protein that is cleaved into a 98-amino-acid signal peptide (SP) and a C-terminal protein (Rem-CT). SP uses retrotranslocation for ER membrane extraction and yet avoids ERAD by an unknown mechanism to enter the nucleus and function as a Rev-like protein. To determine how SP escapes ERAD, we used a ubiquitin-activated interaction trap (UBAIT) screen to trap and identify transient protein interactions with SP, including the ERAD-associated p97 ATPase, but not E3 ligases or Derlin proteins linked to retrotranslocation, polyubiquitylation, and proteasomal degradation of extracted proteins. A dominant negative p97 ATPase inhibited both Rem and SP function. Immunoprecipitation experiments indicated that Rem, but not SP, is polyubiquitylated. Using both yeast and mammalian expression systems, linkage of a ubiquitin-like domain (UbL) to SP or Rem induced degradation by the proteasome, whereas SP was stable in the absence of the UbL. ERAD-associated Derlin proteins were not required for SP activity. Together, these results suggested that Rem uses a novel p97-dependent, Derlin-independent retrotranslocation mechanism distinct from other pathogens to avoid SP ubiquitylation and proteasomal degradation.

## IMPORTANCE

Bacterial and viral infections produce pathogen-specific proteins that interfere with host functions, including the immune response. Mouse mammary tumor virus (MMTV) is a model system for studies of human complex retroviruses, such as HIV-1, as well as cancer induction. We have shown that MMTV encodes a regulatory protein, Rem, which is cleaved into an N-terminal signal peptide (SP) and a C-terminal protein (Rem-CT) within the endoplasmic reticulum (ER) membrane. SP function requires ER membrane extraction by retrotranslocation, which is part of a protein quality control system known as ER-associated degradation (ERAD) that is essential to cellular health. Through poorly understood mechanisms, certain pathogen-derived proteins are retrotranslocated but not degraded. We demonstrate here that MMTV SP retrotranslocation from the ER membrane avoids degradation through a unique process involving interaction with cellular p97 ATPase and failure to acquire cellular proteasome-targeting sequences.

## INTRODUCTION

Protein quality control is critically important for cellular function since misfolding and aggregation are associated with disease states, including Alzheimer’s disease, diabetes, and cancer (1–3). Up to 30% of all cellular proteins are synthesized in association with the endoplasmic reticulum (ER) (4), and yet a large proportion are misfolded and subjected to ER-associated degradation (ERAD) (5). ERAD has multiple components that detect protein misfolding and state of glycosylation (6), including chaperones, glycosidases, and Derlins (3, 7). Derlin-1, -2, and -3 constitute a family of ER transmembrane proteins in mammalian cells that participate in the identification of misfolded proteins for ERAD (8, 9). Following identification, proteins are polyubiquitylated, primarily on lysine residues (10), in a process that requires E1 activating and E2 conjugating enzymes (11) and a set of E3 ligases that are localized to the ER membrane (12). The ubiquitin (Ub) chains on retrotranslocated substrates serve as docking sites for the p97 AAA ATPase and cytosolic proteasomes via various adapter proteins (1, 13). Thus, a variety of different ERAD components are involved in substrate recognition, retrotranslocation, and targeting to the proteasome (1, 14).

Many pathogens use ERAD components to promote their replication and to avoid or suppress the antiviral immune response (15, 16). Viral proteins can serve as ERAD adapters for the disposal of restriction factors and immune signaling molecules that may inhibit viral replication and spread. For example, the human cytomegalovirus (CMV) US2 and US11 proteins target major histocompatibility complex class I (MHC class I) proteins for degradation through ERAD, thus allowing virally infected cells to avoid lysis by cytotoxic T cells (17, 18). Similarly, HIV-1 Vpu protein targets the CD4 viral receptor and BST-2/tetherin for proteasomal degradation through ERAD (17–19). Viruses and bacteria also use ERAD to traffic pathogen-specified proteins out of the ER into the cytosol. For example, *Vibrio cholerae* induces disease through its AB_5_ toxin, which enters the cell by retrograde transport followed by retrotranslocation to the cytosol (20–22). Some viruses, such as simian virus 40 (SV40), use retrograde transport and partial uncoating in the ER to facilitate cell entry (16, 23, 24). Thus, certain pathogens or their products have evolved to subvert ERAD and allow trafficking from the ER to the cytosol.

**FIG 1:**
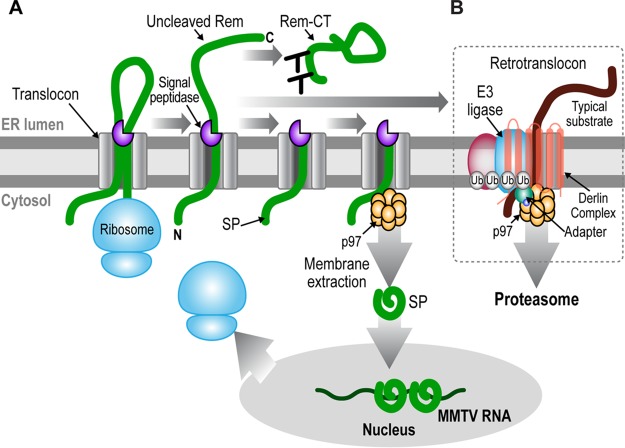
Model for Rem processing and trafficking to allow SP retrotranslocation and evasion of ERAD. (A) Model for Rem processing and trafficking. During translation, exposure of hydrophobic amino acids, including the NES, at the SP C terminus mediate Rem (thick green line) insertion into the ER membrane through the translocon. Completion of translation leads to a type II membrane orientation and cleavage by signal peptidase (purple three-quarter circle) into a membrane-anchored SP and glycosylated Rem-CT. The two glycosylation sites are indicated as Y structures. Uncleaved Rem accumulates in the presence of proteasomal inhibitors, suggesting that it enters the conventional ERAD pathway. Based on data presented here, SP is proposed to interact directly with the p97 ATPase (shown as a gold double-ringed hexamer, indicating the two ATPase domains of each subunit), which extracts SP from the ER membrane into the cytosol. Folded SP then enters the nucleus to bind unspliced MMTV RNA and mediate its export to the cytoplasm through Crm1. (B) Components of the retrotranslocon. Typical ERAD substrates (thick brown line) have folding defects in the luminal, transmembrane, or cytosolic domains. Such defects are recognized by Derlins (the multiple membrane-spanning proteins, which may be homodimers or heterodimers) or by other factors (pink oval), allowing substrate association with one or more of at least 13 different transmembrane E3 ligases that have been linked to ERAD (78). Substrate recognition also involves recruitment of accessory cytosolic factors, such as E1 and E2 enzymes, which initiate addition of polyubiquitin chains (circles labeled Ub), leading to association of other factors, such as Npl4 and Ufd1. These additional factors (small circles labeled as adapters) recognize both the polyubiquitylated substrate and p97 ATPase. Tethering of p97 presumably provides the energy for membrane extraction of many substrates as well as binding of other factors, such as deglycosylating and deubiquitylating enzymes, which are needed prior to substrate delivery to the proteasome for degradation. The exact nature of the retrotranslocon channel is unknown.

ERAD components also are used by mouse mammary tumor virus (MMTV). MMTV is a complex retrovirus that encodes several regulatory and accessory proteins, including Rem (25–27). Rem is a precursor protein that is translated in association with the ER membrane and cleaved by signal peptidase into a 98-amino-acid signal peptide (SP) and a C-terminal fragment (Rem-CT) (26, 28, 29). SP contains an arginine-rich motif (ARM) typical of RNA binding proteins as well as a nuclear/nucleolar localization sequence (NLS/NoLS) and a leucine-rich nuclear export signal (NES) (26), typical of Rev-like nuclear export proteins (30). Steady-state levels of SP are highest in the nucleolus (26), and mutations within the NLS/NoLS prevent SP nucleolar localization and activity in a reporter assay (26). We have shown that a *cis*-acting Rem-responsive element (RmRE) in the reporter vector is necessary for SP-induced activity (31). Furthermore, Rem cleavage is required for SP function in this assay since mutations of the consensus signal peptidase recognition site prevent SP function and localization to the nucleolus (27, 29, 32, 33). SP release from microsomal membranes is independent of signal peptide peptidase (SPP) (29), which affects membrane release of some other viral signal peptides (34–36). Our previous experiments have demonstrated that uncleaved Rem is stabilized by the presence of proteasomal inhibitors, whereas cleaved SP levels are similar in the presence and in the absence of proteasome inhibitors (27). Rem activity appears to require the p97 AAA ATPase (27) (Fig. 1A). These results suggest that SP is extracted from the ER membrane and avoids ERAD, which typically involves polyubiquitylation by an intramembrane E3 ligase and Derlin proteins (Fig. 1B). After extraction, SP serves a Rev-like function for export of MMTV RNA from the nucleus to the cytosol (26–28).

Here, we examine factors that affect retrotranslocation of MMTV-encoded SP into the cytosol prior to nuclear entry using a newly described screen for SP-interacting proteins that depends on covalent linkage to an SP-ubiquitin fusion (ubiquitin-activated interaction trap [UBAIT]). Surprisingly, the p97 ATPase was the only cellular partner identified that was confirmed to be required for SP activity. Using transfection experiments, we detected polyubiquitylation of Rem but not SP. Addition of an N-terminal or C-terminal ubiquitin-like domain (UbL) to SP allowed degradation by purified yeast proteasomes, and transfection experiments in mammalian cells indicated that SP stability was greatly decreased after UbL linkage. Despite the involvement of Derlin proteins in the retrotranslocation of many substrates (23, 37–39), neither Derlin-1 nor Derlin-2 was identified in the UBAIT screen or was needed for SP function by knockdown or knockout approaches in transfection assays. Together, our results suggest a novel p97-dependent and Derlin-independent process for SP retrotranslocation that differs from known pathogen-associated proteins that avoid ERAD.

## RESULTS

### Screen for SP-interacting proteins.

**FIG 2:**
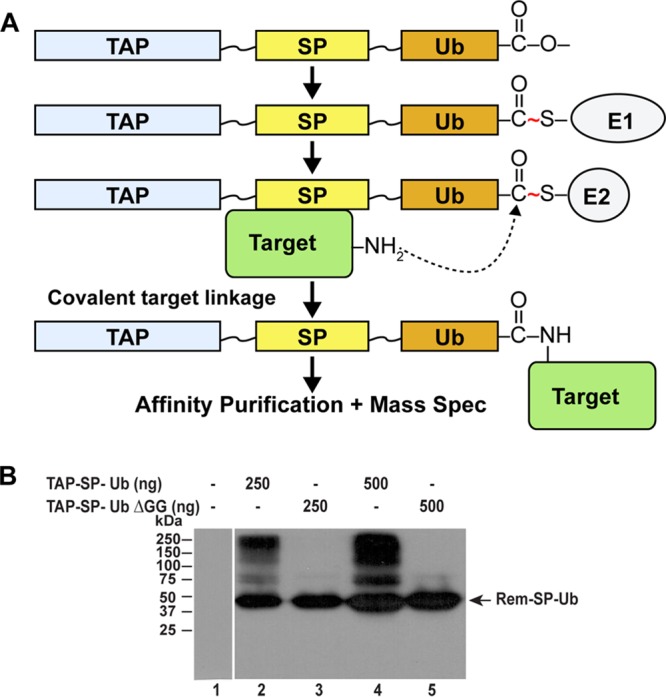
Identification of SP-interacting proteins using UBAITs. (A) Schematic diagram of the UBAIT method. A vector was constructed to express a fusion protein consisting of a purification tag (TAP) at the N terminus and ubiquitin at the C terminus (Ub), separated by flexible linkers from the sequence of a protein of interest (SP). The TAP-SP-Ub expression plasmid was transfected into mammalian cells for expression of the fusion protein, which is activated by an E1 enzyme (either UBA1 or UBA6) and transferred to an E2-conjugating enzyme. The E2 allowed covalent linkage to an associated target protein and affinity purification prior to analysis by mass spectrometry. (B) Western blotting of cells transfected with SP-UBAIT constructs. The TAP-SP-Ub (wild-type ubiquitin at the C terminus) or TAP-SP-UbΔGG (ubiquitin missing the C-terminal glycine residues) expression constructs were transfected into 293 cells (either 250 or 500 ng as indicated). After 48 h, lysates from transfected (lanes 2 to 5) or untransfected (lane 1) cells were used for Western blotting with TAP-specific antibody. The entire experiment was repeated twice.

To determine how SP uses retrotranslocation to perform its Rev-like function in the nucleus, we used the recently described UBAIT method that allows the detection of transient protein interactions (40). This method uses an N-terminal fusion of the tandem affinity purification (TAP) tag and a C-terminal ubiquitin to the protein of interest (SP) (Fig. 2A). Expression of this construct from the cytomegalovirus (CMV) promoter in mammalian cells produces a ubiquitin fusion protein that is activated and conjugated by cellular E1 and E2 enzymes. Proximity of interacting target proteins to the fusion protein then allows their covalent conjugation, and the interacting protein can be identified by TAP and mass spectrometry. Transfection of the fusion construct (TAP-SP-Ub) into 293 cells and Western blotting of cellular extracts with TAP-specific antibody produced multiple bands that migrated more slowly than the fusion protein (Fig. 2B, lanes 2 and 4). Most of these slower-migrating proteins were specific conjugates since transfection of the construct expressing a fusion protein that lacks the C-terminal glycine residues (TAP-SP-UbΔGG) resulted in reduced levels of proteins with a mass greater than that of the fusion protein alone (lanes 3 and 5). Loss of the C-terminal glycines is expected to prevent conjugation of the fusion protein through ubiquitin to interacting proteins (40). Thus, these results suggested that multiple cellular proteins interacted with MMTV-encoded SP.

To identify SP-interacting proteins, larger-scale transfections in 293 cells were performed with individual constructs expressing TAP-SP-Ub or TAP-SP-UbΔGG as well as TAP only or TAP-SP lacking a C-terminal ubiquitin. After 48 h, cellular lysates were prepared and used for affinity purification. Purified material then was subjected to denaturing polyacrylamide gel electrophoresis to separate conjugated and unconjugated TAP-containing proteins. Proteins migrating slower than the expressed protein were excised from the gels and analyzed by mass spectrometry. After performing this experiment twice, multiple proteins were identified as potential SP interactors (for a complete list, see Tables S1 and S2 and combined data in Table S3 in the supplemental material). We considered an interaction specific when at least two-times-greater spectral counts were obtained for proteins associated with TAP-SP-Ub than for TAP only, TAP-SP, or TAP-SP-UbΔGG (Table 1). Only five cellular proteins (p97/VCP, UBA1, UBA6, UBE2O, and USP5) were detected in both purifications and were sufficiently specific by these criteria. Our previous studies also have implicated p97/VCP in Rem function (27). SP interactions with the E1 enzymes UBA1 and UBA6, the E2 enzyme UBE2O, and the deubiquitinase USP5 may be due to their role in the cellular ubiquitylation process, and their detection in our screen may result from the UBAIT method for identification of interacting proteins (40). Strikingly, no Derlin proteins or transmembrane E3 ligases typically associated with ERAD and polyubiquitylated substrates were identified in our screen. Although we cannot exclude the possibility that other ERAD components were missed in our UBAIT screen, we further tested the role of the p97 ATPase and ubiquitylation for SP function.

10.1128/mBio.00328-17.6 TABLE S1  Total proteins identified in the first UBAIT screen. The left-hand columns indicate the identified proteins, the accession number, and the molecular weight. The right-hand columns indicate the number of spectral counts with UBAITs containing TAP only, TAP-SP, TAP-SP-ubiquitin (TAP-SP-Ub), and TAP-SP-ubiquitin lacking the two C-terminal glycine residues (TAP-SP-UbΔGG). Download TABLE S1, XLSX file, 0.02 MB. Copyright © 2017 Byun et al. 2017 Byun et al. This content is distributed under the terms of the Creative Commons Attribution 4.0 International license.

10.1128/mBio.00328-17.7 TABLE S2  Total proteins identified in the second UBAIT screen. The left-hand columns indicate the identified proteins, the accession number, and the molecular weight. The right-hand columns indicate the number of spectral counts with UBAITs containing TAP only, TAP-SP, TAP-SP-ubiquitin (TAP-SP-Ub), and TAP-SP-ubiquitin lacking the two C-terminal glycine residues (TAP-SP-UbΔGG). Download TABLE S2, XLSX file, 0.03 MB. Copyright © 2017 Byun et al. 2017 Byun et al. This content is distributed under the terms of the Creative Commons Attribution 4.0 International license.

10.1128/mBio.00328-17.8 TABLE S3  Combined proteins identified using two UBAIT screens. Columns are indicated as in Tables S1 and S2. Proteins colored in blue have the specificity of SP-interacting proteins. Proteins colored in pink have the specificity of SP-interacting proteins and have been implicated in ERAD. Proteins colored in yellow were identified in both screens but did not have the specificity expected for SP-interacting proteins. Proteins that were not detected in both screens are presented without additional color. Download TABLE S3, XLSX file, 0.03 MB. Copyright © 2017 Byun et al. 2017 Byun et al. This content is distributed under the terms of the Creative Commons Attribution 4.0 International license.

**TABLE 1:** SP interaction candidates identified by the UBAIT method

Candidate protein	Spectral counts^a^			
	TAP	TAP-SP	TAP-SP-Ub	TAP-SP-UbΔGG
p97/VCP	8, 7	6, 5	44, 36	7, 5
UBA1	9, 6	4, 4	40, 33	6, 4
USP5	3, 4	1, 1	34, 41	0, 0
UBE2O	0, 0	1, 3	20, 11	1, 4
UBA6	1, 0	0, 1	7, 13	0, 0

^a^ Spectral counts identified with each transfected construct by mass spectrometry. Counts of two separate transfections and TAP-based purifications are separated by a comma.

### DN ATPase p97 suppresses SP activity.

**FIG 3:**
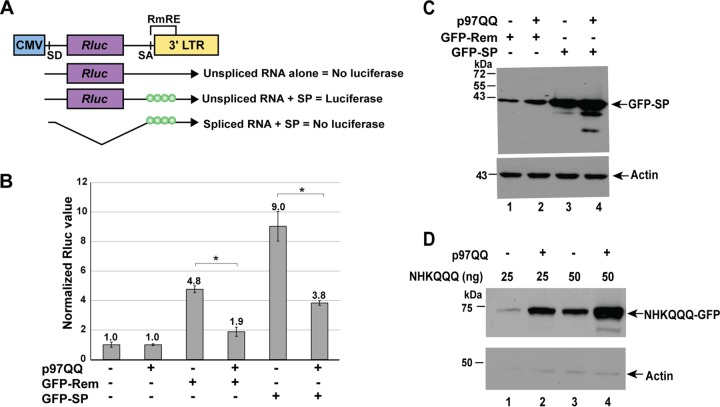
A dominant negative AAA ATPase p97 inhibits the activity of both Rem and SP. (A) Reporter assay to detect MMTV-encoded SP activity. The *Renilla* luciferase gene was inserted between a splice donor (SD) and a splice acceptor (SA) site at the 3′ end of the MMTV genome. Expression of unspliced reporter RNA in the absence of Rem or splicing of the reporter RNA in the presence of Rem yields no luciferase activity. Rem expression in the presence of the unspliced reporter RNA allows binding to the Rem-responsive element (RmRE) and dose-dependent luciferase expression. LTR, long terminal repeat. (B) DN p97 expression inhibits the activity of GFP-tagged Rem and SP. The means ± standard deviations of luciferase values of triplicate transfections in 293 cells are shown. *Renilla* luciferase activity from the pHM*Rluc* Rem-responsive plasmid was determined after normalization for transfection efficiency using the activity of a cotransfected Rem-nonresponsive plasmid (pGL3-control). Values in the presence or absence of DN p97 (p97QQ) were assigned a relative value of 1.0 and compared to the activity after expression of GFP-Rem or GFP-SP. Rem and SP activity was statistically lower in the presence of p97QQ (as evaluated by a two-tailed Student *t* test). (C) Both GFP-Rem and GFP-SP are expressed in the presence of p97QQ. Extracts from the experiment in panel A were subjected to Western blotting with GFP- or actin-specific antibodies. (D) A control ERAD substrate, NHKQQQ, accumulates in the presence of DN p97QQ. Transfections of NHKQQQ expression plasmids were performed in the presence and absence of DN p97QQ. Extracts from transfected cells were subjected to Western blotting using GFP- or actin-specific antibodies. This experiment was done at least twice.

Our previous results indicated that Rem, which accumulates in the presence of proteasome inhibitors, is cleaved by signal peptidase to yield SP and Rem-CT. SP levels were relatively unaffected by proteasomal inhibition, but Rem function required p97 AAA ATPase activity (27). SP, which is expressed from both Rem and Env (27), may not require p97-mediated extraction after cleavage since the proposed signal recognition particle binding site is located at the C terminus of SP (29). To directly test whether SP expression alone requires p97 activity, we cotransfected 293 cells with constructs expressing an N-terminally green fluorescent protein (GFP)-tagged full-length Rem (GFP-Rem) or SP (GFP-SP) with the reporter plasmid pHM*Rluc* (26) in the presence and absence of a plasmid encoding a dominant negative (DN) form of p97 (p97QQ) (41). The luciferase activity of the pHM*Rluc* plasmid has been shown to be a sensitive and specific indicator of MMTV SP function. SP binding to the RmRE on the reporter RNA allows export of unspliced transcripts from the nucleus and cytosolic translation of *Renilla* luciferase proportional to SP levels (Fig. 3A) (26, 31). As expected, the transfection of constructs expressing GFP-Rem yielded ~5-fold-increased levels of *Renilla* luciferase activity, and this induction was inhibited 2.5-fold by DN p97 ATPase (p97QQ) (Fig. 3B) (27). Similarly, GFP-SP expression induced pHM*Rluc* reporter activity by 9-fold (32), and this induction was inhibited 2.4-fold by the DN p97. Western blotting confirmed that SP was produced from both vectors, although the Rem expression vector yielded smaller amounts of SP, presumably due to increased ERAD of this precursor (27). The presence of the DN p97 did not alter SP cleavage (Fig. 3C). We also cotransfected DN p97 with a control construct expressing the nonglycosylated null Hong Kong mutant of α-1 antitrypsin (NHKQQQ), which is a known substrate of ERAD (42, 43). As expected, DN p97 blocked retrotranslocation of NHKQQQ, causing its accumulation (Fig. 3D). Thus, inhibition of GFP-SP activity by DN p97 implies that SP is cotranslationally inserted into the ER membrane without associated synthesis of the Rem C terminus or Env and requires p97 for its extraction into the cytosol prior to MMTV-reporter RNA binding in the nucleus.

### SP polyubiquitylation is not detectable.

**FIG 4:**
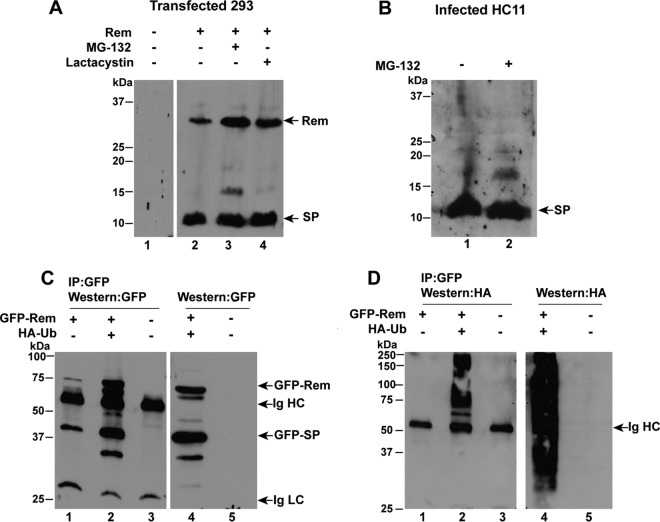
MMTV-encoded SP polyubiquitylation is undetectable prior to retrotranslocation. (A) Treatment of Rem-transfected cells with proteasome inhibitors fails to detect polyubiquitylated SP. Cells (293) were transfected with untagged Rem and treated with either MG-132 or lactacystin for 12 h as indicated. Extracts obtained 48 h posttransfection were used for Western blotting with SP-specific antibodies. The positions of Rem and cleaved SP are shown by arrows. (B) MMTV-producing mammary cells have no detectable ubiquitylated Rem or SP in the presence of proteasome inhibitor. HC11 mammary cells infected with MMTV were grown in the presence or absence of MG-132 for 12 h, and extracts were prepared. Western blots with SP-specific antibody are shown. Results in panels A and B are similar to previously published results (27, 32). (C) Immunoprecipitations of GFP-tagged Rem in the presence of HA-tagged ubiquitin with GFP-specific antibodies. Cells (293) were transfected with GFP-tagged Rem in the presence and absence of HA-tagged ubiquitin. Extracts prepared 48 h posttransfection were either immunoprecipitated with GFP-specific antibodies (lanes 1 to 3) or used directly for Western blotting with GFP-specific antibodies (lanes 4 and 5). The positions of GFP-tagged Rem and SP as well as heavy and light chains of immunoglobulin (Ig HC and Ig LC, respectively) are indicated by arrows. IP, immunoprecipitation. (D) Detection of HA-ubiquitylated forms of GFP-Rem. The same samples used in panel C were subjected to Western blotting with HA-specific antibodies. Immunoprecipitations were performed at least twice.

Previous experiments indicated that uncleaved Rem is stabilized in the presence of proteasome inhibitors, whereas, after cleavage, SP levels are relatively unaffected by such inhibitors (27, 32). We now tested whether ubiquitylated forms of Rem or SP were detectable. HEK 293 cells were transfected with a Rem expression vector in the presence or absence of two different proteasome inhibitors, MG-132 and lactacystin. Consistent with our published results (27, 32), Western blotting assays using SP-specific antibody showed that levels of the uncleaved Rem of 33 kDa, but not SP, increased in the presence of either inhibitor (Fig. 4A). A faint band of ~16 kDa was detected in the presence of MG-132 (lane 3) but was not observed consistently or with the more specific lactacystin inhibitor and may be due to stabilization of a Rem breakdown product. Nevertheless, a ladder of polyubiquitylated SP was not observed.

To determine whether polyubiquitylated Rem was detectable in MMTV-infected cells, we also tested HC11 mouse mammary epithelial cells that expressed a cloned infectious MMTV provirus (pHYB-MTV) (44, 45) (Fig. 4B). Western blotting assays of extracts using SP-specific antibody showed a strong band of ~11 kDa, which did not intensify when extracts were prepared from cells grown in the presence of MG-132 (compare lanes 1 and 2). Interestingly, Rem was not detectable with or without proteasomal inhibitors, although the major source of SP in MMTV-infected cells is due to cleavage of the more abundant envelope protein rather than Rem (26, 27). Although we cannot eliminate the possibility that a deubiquitinating enzyme acts selectively on SP, these results suggested that SP was not polyubiquitylated.

To test for SP polyubiquitylation more conclusively, we used a more sensitive method. Constructs expressing GFP-tagged Rem were transfected into 293 cells in the presence or absence of a vector expressing hemagglutinin (HA)-tagged ubiquitin. Lysates from transfected cells were then subjected to immunoprecipitation with GFP-specific antibody, and precipitates were analyzed by Western blotting using antibodies specific for GFP or HA. The levels of GFP-Rem and GFP-SP increased in the presence of HA-ubiquitin, but polyubiquitylated forms of these proteins were not apparent using GFP-specific antibody (Fig. 4C, lanes 2 and 4). Further, Western blotting assays using HA-specific antibody detected a variety of HA-ubiquitylated proteins in lysates and immunoprecipitates. As expected, HA-ubiquitylated forms larger than Rem were observed after immunoprecipitation (Fig. 4D, lane 2) as well as a smear of ubiquitylated products from whole-cell lysates (Fig. 4D, lane 4). Ubiquitylated forms of GFP-tagged SP were not observed between the 37- and 50-kDa markers. These results are consistent with Rem polyubiquitylation prior to signal peptidase cleavage (27) but resistance of cleaved SP to ubiquitin addition after Rem cleavage. Therefore, lack of SP ubiquitylation after p97 extraction from the ER membrane likely prevents its targeting for proteasomal degradation.

### SP can be degraded by purified yeast proteasomes.

Previous experiments with different cellular substrates have indicated that proteasomal degradation requires a degradation signal or degron, which is recognized by E3 ligases that generate polyubiquitin chains on amino acids (typically lysines) within the target protein (46, 47). The proteasome binds its target proteins at the ubiquitin tag but initiates degradation at a disordered region within the target (48–51). Thus, SP could employ at least two strategies to escape degradation: avoiding ubiquitylation or preventing proteasome initiation.

**FIG 5:**
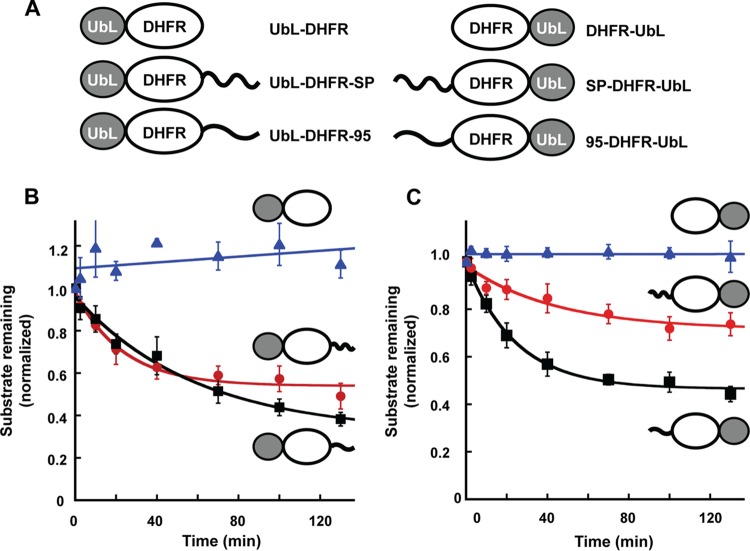
MMTV-encoded SP is degraded by yeast proteasomes. (A) Diagram of DHFR constructs tested for degradation by purified yeast proteasomes. Constructs encoded proteins with a UbL domain (dark oval) at either the N-terminal or C-terminal end of DHFR in the absence of a tail or the 95-amino-acid tail derived from *S. cerevisiae* cytochrome *b*_2_ (50) or MMTV SP. (B) Time-dependent degradation of N-terminally UbL-tagged DHFR proteins by purified yeast proteasomes. Symbols: blue triangles, UbL-DHFR; red circles, UbL-DHFR-SP; black squares, UbL-DHFR-95. (C) Time-dependent degradation of C-terminally UbL-tagged DHFR proteins by purified yeast proteasomes. This experiment was repeated at least four times. Symbols: blue triangles, DHFR-UbL; red circles, SP-DHFR-UbL; black squares, 95-DHFR-UbL.

To test whether the proteasome is able to recognize and degrade the lysine-rich MMTV-encoded SP when targeted for destruction, we engineered a UbL from Rad23 at the N terminus of *Escherichia coli* dihydrofolate reductase (DHFR) to allow proteasome binding as previously described (49, 50, 52) (UbL-DHFR, Fig. 5A). This “tailless” construct also was modified by the addition of SP at the C terminus of DHFR (UbL-DHFR-SP) or a known 95-amino-acid-long proteasome initiation site (UbL-DHFR-95). Each of the substrates was radioactively labeled in an *in vitro* transcription-translation reaction, partially purified, and incubated with immunoaffinity-purified yeast proteasomes (Fig. 5B). The amount of degradation was assessed on sodium dodecyl sulfate (SDS)-containing polyacrylamide gels using quantitative autoradiography. As predicted from previous results (49, 50), no substrate degradation was detectable in the absence of a C-terminal tail. Addition of the SP tail enabled proteasomal degradation of UbL-DHFR-SP with kinetics similar to the positive-control protein (UbL-DHFR-95) (50, 51). Equivalent experiments also were performed using constructs where SP was added to the N terminus of DHFR and a UbL domain was added at the C terminus (SP-DHFR-UbL) (Fig. 5C). Similarly to the UbL-DHFR protein, the tailless DHFR-UbL protein was not degraded by yeast proteasomes, whereas addition of the 95-amino-acid initiation sequence to the N terminus gave efficient degradation (Fig. 5C). N-terminal addition of SP to DHFR-UbL induced degradation, but with slower kinetics and to a lower extent than the 95-amino-acid tail. These experiments suggested that SP contains an unstructured region that allows the yeast proteasome to initiate degradation when the protein is targeted to the proteasome by a UbL domain.

### SP lacks a proteasome degradation signal in mammalian cells.

**FIG 6:**
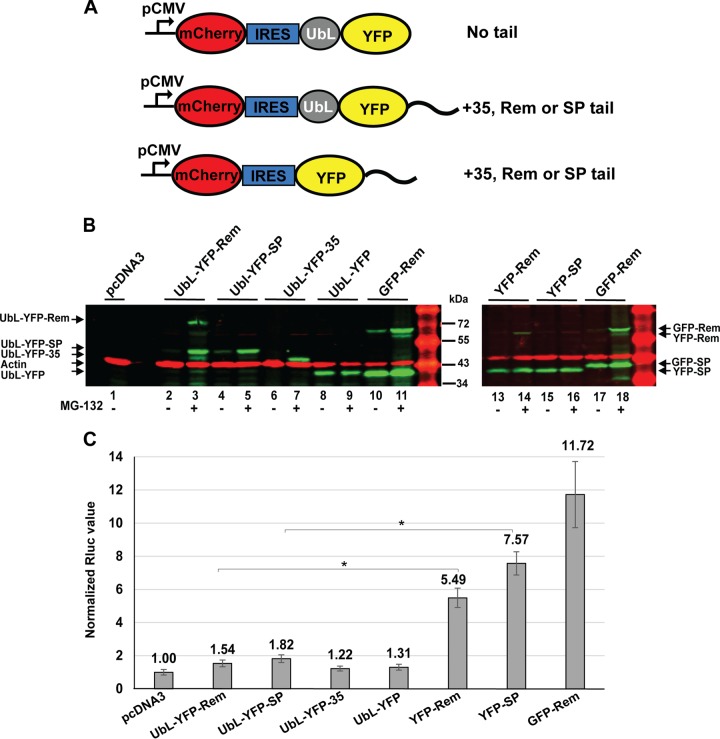
Addition of a UbL domain allows degradation of SP in mammalian cells. (A) Diagram of constructs used to test the degradation of YFP in mammalian cells. Constructs contained a CMV promoter for expression of a bicistronic transcript that is translated into mCherry at the 5′ end using cap-dependent translation. YFP with or without an N-terminal UbL was expressed from the 3′ end of the transcript by IRES-mediated translation. Expressed proteins had either no tail, a 35-amino-acid tail derived from cytochrome *b*_2_, or a tail from Rem or SP as indicated. (B) Western blotting of cells transfected with UbL-YFP-expressing constructs. Cells were transfected with 500 ng of YFP-tagged expression plasmids or 50 ng of GFP-Rem expression plasmid. Extracts from triplicate transfections grown in the presence (lanes 3, 5, 7, 9, 11, 14, 16, and 18) and absence (all other lanes) of MG-132 were subjected to Western blotting and incubation with antibody to GFP, which detects both YFP and GFP, or with actin-specific antibodies. Appropriate secondary antibodies were used prior to LI-COR analysis. (C) Decreased SP activity after fusion with a UbL domain. Cells were transfected with 50 ng of YFP-tagged expression plasmids or 12.5 ng of GFP-Rem expression plasmid (positive control) with the addition of the reporter plasmids pHM*Rluc* and pGL3-control. Luciferase activities normalized for transfection efficiency are reported as the means ± standard deviations from triplicate transfections; statistical analyses were performed with a two-tailed Student *t* test. The reporter activity of transfections lacking fusion protein constructs (replaced with pcDNA3 control DNA) was assigned a relative value of 1.0. This experiment was repeated twice.

To assess the ability of SP to be degraded by the proteasome in mammalian cells after addition of a UbL, we prepared another set of constructs that expressed a yellow fluorescent protein (YFP) from a cytomegalovirus (CMV) promoter (Fig. 6A). Individual YFP expression plasmids were then modified by the addition of different C-terminal tails. Each of the constructs was transfected into 293 cells and analyzed for expression of the fluorescent proteins. Although mCherry was inserted followed by an internal ribosome entry site (IRES) at the 5′ end of each construct to act as a control for transfection efficiency, we observed that the degradation of the downstream YFP also affected mCherry expression by an unknown mechanism. Therefore, we analyzed the expression of each construct in transfected cells directly by SDS-polyacrylamide gel electrophoresis and Western blotting.

Western blotting assays using GFP-specific antibodies, which also detected the related YFP fusions, showed that YFP-Rem was cleaved to yield YFP-SP as shown for the control GFP-Rem protein (Fig. 6B, compare lanes 13 and 17). Similarly to published results (27), uncleaved Rem was greatly stabilized by MG-132, whereas the levels of cleaved SP were relatively independent of the presence of the proteasomal inhibitor (compare lanes 13 and 14 or lanes 17 and 18). Attachment of a UbL domain to the N terminus of YFP-SP alone or YFP-Rem (yielding YFP-SP through cleavage) led to their degradation. Addition of the UbL to SP was as effective as that observed for the positive-control UbL-YFP-35 (Fig. 6B, lanes 2, 4, and 6). Addition of the proteasome inhibitor MG-132 rescued all UbL-containing fluorescent protein fusions (Fig. 6B, lanes 3, 5, and 7), indicating that the UbL fusions were degraded by the proteasome. UbL-YFP lacking any tail was stable even in the absence of proteasome inhibitors (Fig. 6B, lanes 8 and 9). Thus, the proteasome was able to initiate degradation using SP sequences. However, YFP-SP lacking the UbL domain was not degraded by the proteasome because the presence of MG-132 did not affect its abundance (Fig. 6B, lanes 15 and 16), suggesting the absence of a proteasome degradation signal within SP. These results are consistent with the conclusion that SP is not polyubiquitylated but does not resist degradation when targeted to the proteasome.

To determine whether functional SP was generated from YFP fusion proteins, we transfected YFP expression constructs into HEK 293 cells in the presence of the pHM*Rluc* reporter plasmid (26). The assay is highly sensitive for the detection of SP activity as described previously (28, 31), and the transfection of 12.5 ng of the GFP-Rem expression plasmid gave ~12-fold-higher *Renilla* luciferase levels than the pcDNA3 control (Fig. 6C). Plasmids lacking Rem sequences (UbL-YFP-35 and UbL-YFP) also had no detectable activity. The YFP-Rem and YFP-SP constructs showed ~5.5-fold- and 7.6-fold-increased reporter levels, respectively, after transfection of 50 ng of plasmid DNA, which was in the linear range of the assay (Fig. S1). These results indicated that the YFP-Rem expression plasmid had approximately 10-fold-lower activity than the comparable GFP-Rem expression construct, presumably due to differences in the linker between the fluorescent protein and Rem. Expression of UbL-tagged forms of YFP-Rem or YFP-SP had little increase in activity over that observed with UbL-YFP control plasmids lacking SP. This result may be due to UbL domain targeting of YFP-Rem constructs for degradation, thus preventing SP accumulation. In agreement with previous results (26), the constructs expressing SP generated higher reporter levels than those expressing Rem, presumably because Rem is ubiquitylated. Therefore, both the Western blotting and reporter assays suggested that the proteasome has an intrinsic ability to initiate SP degradation and that SP stability reflects the fact that it is not ubiquitylated and directed to the proteasome.

10.1128/mBio.00328-17.1 FIG S1  Titration of SP-containing plasmids in the pHM*Rluc* reporter assay. HEK 293 cells were transfected with Rem-responsive and -nonresponsive plasmids in the presence of the control pcDNA vector alone or with YFP-Rem, YFP-SP, or GFP-Rem in the indicated amounts. Results are reported as described in Materials and Methods. The data demonstrate that this experiment was performed in the linear range of the assay. Statistical analysis was performed with a two-tailed Student *t* test. This experiment was repeated twice. Download FIG S1, TIF file, 0.2 MB. Copyright © 2017 Byun et al. 2017 Byun et al. This content is distributed under the terms of the Creative Commons Attribution 4.0 International license.

### Derlin-1 is not required for SP retrotranslocation.

**FIG 7:**
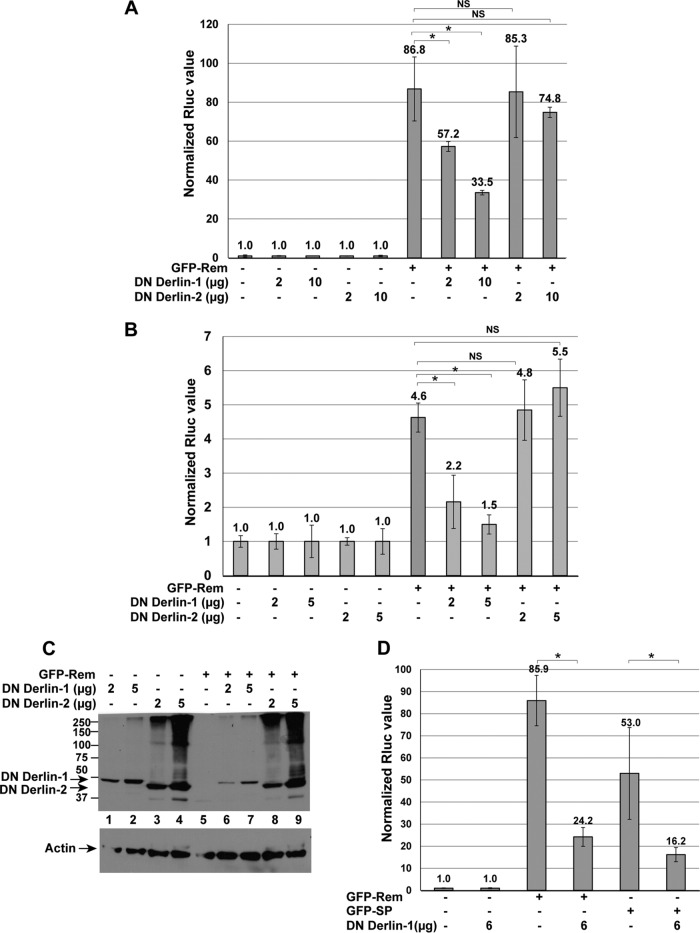
Dominant negative Derlin-1 inhibits Rem activity. (A) DN Derlin-1, but not DN Derlin-2, inhibits Rem activity in mouse mammary cells. HC11 cells were transfected by electroporation and analyzed as described in Materials and Methods. Two different amounts of the Derlin expression plasmids were transfected as indicated. All samples contained the same total amounts of DNA. Each value obtained in the absence of Rem, but containing the two reporter vectors, was normalized to a relative value of 1.0. (B) DN Derlin-1, but not DN Derlin-2, inhibits Rem activity in human HEK 293 cells. Cells (293) were transfected with two different amounts of the Derlin expression vectors as indicated. Luciferase values were normalized to 1.0 in the absence of the Rem expression vector. Statistical analysis was performed by a two-tailed Student *t* test. (C) Western blotting confirms expression of DN Derlin proteins. Extracts derived from 293 cells transfected with the indicated constructs in the presence of reporter plasmids were used for Western blotting and incubation with actin-specific antibody or a GFP antibody, which also detects YFP. GFP-Rem and GFP-SP are not observed due to the low level of these transfected plasmids (12.5 ng). Numbers at left are molecular masses in kilodaltons. (D) The activity of both Rem and SP is inhibited by the presence of DN Derlin-1. Transfections of reporter and expression constructs were performed in HC11 cells, and results of reporter assays are reported as described in Materials and Methods. Statistical analysis was performed by a two-tailed Student *t* test. This experiment was performed three times.

Derlin proteins are known to be involved in ERAD of many substrates (9, 12, 37). Mammals have three functional Derlin proteins, Derlin-1, -2, and -3, although Derlin-3 is expressed in only a limited number of tissues (8). Since Rem is active in a variety of cell types (26, 31), we determined whether Derlin-1 or Derlin-2 affected Rem retrotranslocation. HC11 mouse mammary cells were transfected with Rem and reporter constructs in the presence or absence of plasmids expressing DN Derlin-1 or DN Derlin-2 (Fig. 7A). Each DN protein is a fusion to a C-terminal YFP tag (53). As expected, reporter gene activity was induced by ~90-fold by Rem (lane 6). Neither DN protein significantly decreased reporter activity in the absence of Rem, and yet expression of increasing amounts of DN Derlin-1 inhibited Rem function in a statistically significant manner (up to ~2.5-fold in these experiments), whereas a DN Derlin-2 did not. This experiment also was repeated in 293 cells with similar results (Fig. 7B). Western blotting of these cell extracts for the DN proteins revealed that the Derlin-2 fusion protein was expressed at higher levels than the Derlin-1 fusion (Fig. 7C), suggesting that failure of DN Derlin-2 to affect SP activity was not simply due to lower DN Derlin-2 expression. We also confirmed that DN Derlin-2 was functional in our assay since coexpression of DN Derlin-2 increased the stability of a known substrate, NHK-GFP (54) (Fig. S2). Thus, DN Derlin-1, but not DN Derlin-2, expression appears to inhibit Rem activity, presumably during retrotranslocation from the ER membrane to the cytosol.

10.1128/mBio.00328-17.2 FIG S2  NHK-GFP accumulates in the presence of DN Derlin-2. NHK is a well-known ERAD substrate that is dependent on Derlin-2 for retrotranslocation. C-terminally GFP-tagged NHK was used as a positive control for the activity of the DN Derlin-2 construct. NHK-GFP (50 ng) and DN Derlin-2 (5 µg) were cotransfected into HEK 293 cells. Cell extracts were prepared 48 h posttransfection and used for Western blotting. Blots were incubated with antibodies specific for GFP and actin and developed according to the LI-COR method. This experiment was performed at least three times. Download FIG S2, TIF file, 0.5 MB. Copyright © 2017 Byun et al. 2017 Byun et al. This content is distributed under the terms of the Creative Commons Attribution 4.0 International license.

In the SP expression vector, the signal sequence ends with the translation termination codon, which may disable the ER targeting mechanism and allow SP to escape the ribosome and enter the cytosol without trafficking through the ER membrane. To determine whether Derlin-1 is required for SP retrotranslocation (i.e., when the C terminus of Rem is absent), we cotransfected either GFP-Rem or GFP-SP expression constructs into HC11 cells with or without plasmids expressing DN Derlin-1 (Fig. 7D). DN Derlin-1 protein inhibited the activity of GFP-Rem as well as GFP-SP lacking Rem-CT. Although our SP-UBAIT screen did not detect interaction with Derlin-1 (Table 1), these results suggested that expression of DN Derlin-1 inhibited efficient dislocation of SP from the ER membrane in the presence or absence of the C terminus.

**FIG 8:**
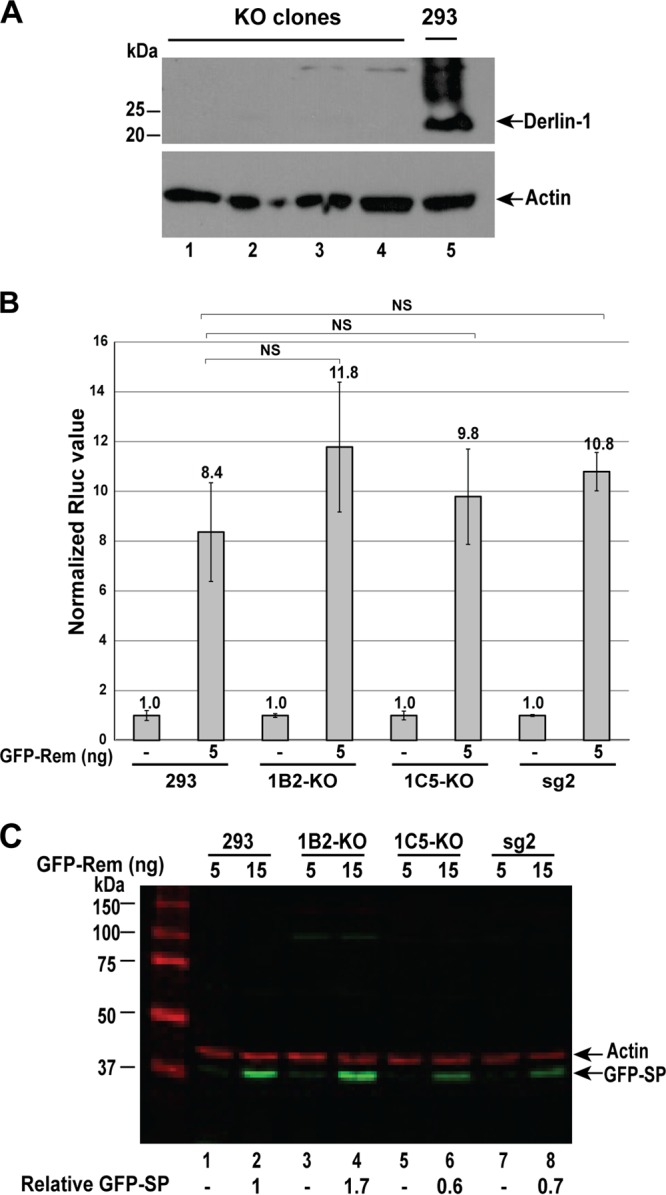
CRISPR-Cas knockout of Derlin-1 does not affect MMTV SP activity. (A) Western blotting confirms Derlin-1 knockout in 293 cells. Extracts were obtained from four different clonal cell lines obtained after transfection with two Derlin-1-specific guide RNAs. Lanes 1 to 4 contain extracts from clones 1B1, 2B4, 1B2, and 1C5, whereas lane 5 contains an extract from the parental 293 cell line. Blots were incubated with antibodies specific for Derlin-1 (top) or actin (bottom). (B) SP function is unaltered in Derlin-1-knockout cells. Derlin-1-expressing 293 cells, two different knockout clones (1B2 and 1C5), and a Derlin-1-expressing clone selected with a single guide RNA, which was insufficient for knockout, were analyzed. Each cell line was transfected in triplicate with the indicated amount of GFP-Rem expression plasmid and the Rem-responsive pHM*Rluc* plasmid, as well as a Rem-nonresponsive plasmid (pGL3-control), to normalize for transfection efficiency. After 48 h, extracts were assayed for *Renilla* and firefly luciferase activities. Results are reported as described in Materials and Methods. The normalized values for 5 ng of GFP-Rem expression plasmid in two different knockout clones were not statistically different (analyzed by a two-tailed Student *t* test) from those obtained with the parental 293 cells or a clone treated with a single guide RNA expressing Derlin-1 (sg2). (C) Rem is processed normally in Derlin-1-knockout cells. Extracts from additional samples in the experiment in panel B were used for Western blotting with GFP and actin-specific antibodies and quantitated using the LI-COR method. Values of GFP-SP expression were normalized relative to that of the parental 293 cells. This experiment was repeated in multiple clones as shown.

DN Derlin-1 expression may have indirect effects since knockdown of Derlin-1 with siRNA and expression of Derlin-1-YFP had different effects on retrotranslocation of cholera toxin (53, 55). To test more directly for the role of Derlin-1 in SP retrotranslocation, we used CRISPR-Cas (clustered regularly interspaced short palindromic repeat) technology to disrupt the Derlin-1 gene in 293 cells. To minimize potential alterations of nontargeted genes (56), the Cas9 D10A mutation was introduced to allow DNA nickase activity in combination with two different guide RNAs (gRNAs) specific for the *DERL1* gene. Lentivirus vectors expressing the mutant Cas9 and the specific guide RNAs were transfected into 293 cells and selected for stable vector expression. As previously shown by others (38), 293 cells lacking functional Derlin-1 were viable since Western blotting confirmed that all of these clones lacked expression of Derlin-1 (Fig. 8A). Two different Derlin-1-knockout clones were then transfected with Rem expression plasmids in the presence of the reporter pHM*Rluc* (26, 28). The results obtained from distinct clonal knockout lines indicated no significant difference in SP function in the presence and absence of Derlin-1 (Fig. 8B). Two additional clones also were tested for activity in the reporter assay with similar results. Differences in GFP-SP expression reflected different transfection efficiencies of individual clones as determined by the values of the Rem-nonresponsive pGL3-control reporter activity (see Fig. S3). Western blotting confirmed that Rem is processed normally in the absence of Derlin-1 (Fig. 8C). Therefore, in contrast to transfections using vectors expressing the DN Derlins, the results of transfections of wild-type and Derlin-1-knockout cell lines indicate that SP retrotranslocation does not require Derlin-1.

10.1128/mBio.00328-17.3 FIG S3  *DERL1*-knockout clones have different transfection efficiencies. Two different *DERL1*-knockout cell clones (1B2 and 1C5) were transfected with 5 ng of GFP-Rem expression vector as indicated in the presence of the Rem-responsive plasmid pHM*Rluc* (expressing *Renilla* luciferase) and the Rem-nonresponsive pGL3-control (expressing firefly luciferase). The results of *Renilla* (A) and firefly (B) luciferase assays are plotted separately and compared to the results in the parental 293 cells or cells selected in the presence of a single guide RNA (sg2), both of which had wild-type levels of Derlin-1. After normalization for transfection efficiency, the knockout of *DERL1* had no effect on SP activity (Fig. 8). Statistical analysis was performed with a two-tailed Student *t* test. The same experiment was repeated in multiple clones with the same result. Download FIG S3, TIF file, 0.2 MB. Copyright © 2017 Byun et al. 2017 Byun et al. This content is distributed under the terms of the Creative Commons Attribution 4.0 International license.

To further examine the role of Derlins in SP function, we tested the level of expression of Derlin-2 and Derlin-3 in the Derlin-1-knockout cells. The wild-type 293 cells and two different Derlin-1-knockout clones were used for lysate preparation and Western blotting with Derlin-2-specific antibody. The results suggested that Derlin-2 is slightly overexpressed in the knockout cells relative to the parental 293 cells (Fig. S4A), perhaps to compensate for Derlin-1 loss. In contrast, Derlin-3 was not expressed in the wild-type or Derlin-1-knockout cells but was easily detected in splenic cell extracts (Fig. S4B). To determine whether DN Derlin-2 could affect SP activity in the absence of Derlin-1, expression vectors for DN Derlin-2 and GFP-Rem were cotransfected into Derlin-1-knockout cells. No statistical difference in SP activity was observed in the presence and absence of DN Derlin-2 (Fig. S5A) when both Rem cleavage and dominant negative protein expression were verified by Western blotting (Fig. S5B). Thus, in agreement with our initial screen for SP-interacting proteins, these experiments indicate that SP retrotranslocation and function are Derlin independent.

10.1128/mBio.00328-17.4 FIG S4  Expression of Derlin-2 and Derlin-3 in HEK 293 cells and *DERL1*-knockout clones. (A) Expression of Derlin-2 in *DERL1*-knockout cells. Extracts from HEK 293 parental and *DERL1*-knockout cells were used for Western blotting. Blots were incubated with antibodies specific for Derlin-2 (top) or actin (bottom). (B) Derlin-3 is not expressed in the knockout or parental 293 cells. Derlin-3 expression in a lysate from a BALB/c mouse spleen served as a positive control. Blots were incubated with antibodies specific for Derlin-3 (top) or actin (bottom). The experiment was performed twice. Download FIG S4, TIF file, 0.2 MB. Copyright © 2017 Byun et al. 2017 Byun et al. This content is distributed under the terms of the Creative Commons Attribution 4.0 International license.

10.1128/mBio.00328-17.5 FIG S5  Dominant negative Derlin-2 has no effect on Rem activity in *DERL1*-knockout cells. (A) DN Derlin-2 does not inhibit Rem activity in human parental HEK 293 and derived Derlin-1-knockout cells. HEK 293 and Derlin-1-knockout (1C5) cells were cotransfected with expression vectors for GFP-Rem (12.5 ng), pHM*Rluc* (250 ng), pGL3-control (250 ng), and Derlin-2 (2 µg). Luciferase values were normalized to 1.0 in the absence of the Rem expression vector as described in Materials and Methods. Statistical analysis was performed with a two-tailed Student *t* test. (B) Western blotting confirms expression of DN Derlin-2 and GFP-SP proteins. Extracts derived from *DERL1*-knockout (1C5) cells transfected with the indicated constructs in the presence of reporter plasmids were used for Western blotting. Blots were incubated with GFP antibody. This experiment was performed at least twice. Download FIG S5, TIF file, 0.4 MB. Copyright © 2017 Byun et al. 2017 Byun et al. This content is distributed under the terms of the Creative Commons Attribution 4.0 International license.

## DISCUSSION

Rem is a precursor protein involved in the nuclear export of unspliced MMTV RNA as well as postexport functions that are dependent on the *cis*-acting Rem-responsive element found in all known MMTV mRNAs (25, 26, 31, 57). This retroviral regulatory protein has an unusual trafficking scheme in which the N-terminal SP mediates cotranslational insertion into the ER membrane. Rem is subsequently cleaved by translocon-associated signal peptidase into SP and a C-terminal product (Rem-CT) (27, 29, 31). Following cleavage, SP is retrotranslocated into the cytosol prior to nuclear entry and binding to viral RNA (27, 32). Membrane extraction of MMTV-encoded SP is independent of cleavage by SPP or SPP-like enzymes (29), unlike other viral and cellular signal peptides (34–36). In this study, we used the UBAIT method to detect cellular proteins that associate with SP even transiently. In two biological replicates, only p97, UBA1, UBA6, USP5, and UBE2O were specifically conjugated to the TAP-SP-Ub fusion protein (Table 1). Of these candidates, UBA1, UBA6, UBE2O, and USP5 likely are the result of the UBAIT method since UBA1 and UBA6 are identified in every screen and UBE2O and USP5 often are identified regardless of the ubiquitin fusion protein used (40). We established that SP synthesized in the absence of Rem-CT requires the AAA ATPase p97 for retrotranslocation (Fig. 3) as previously demonstrated for Rem (27). Moreover, the UBAIT method, which is based on covalent linkage to a ubiquitin fusion protein, together with our other results suggests a direct association between p97 and SP.

Rem appears to be polyubiquitylated and subjected to ERAD since increased levels are detected in the presence of the proteasome inhibitor MG-132 or lactacystin (27). In contrast, SP levels were only slightly affected by these inhibitors (Fig. 4), suggesting that SP is not ubiquitylated, despite being rich in lysine residues (25, 26). Coexpression of Rem with HA-tagged ubiquitin detects a broad smear of labeled products, and yet immunoprecipitation detected only ubiquitylated proteins that are larger than Rem, suggesting that full-length Rem, but not cleaved SP, is the target of an E3 ligase.

Prototypical proteasome substrates have been studied in both yeast and mammalian cells (48–52). These experiments indicate that polyubiquitin chains are recognized by the proteasome, but that an unstructured region is needed to allow the proteasome to engage its substrate and initiate degradation (48, 49). Addition of SP as a C-terminal tail to a stable DHFR substrate with an N-terminal UbL showed that SP is recognized and degraded by purified yeast proteasomes (Fig. 5). In contrast, SP at the N terminus of DHFR with a UbL at the C terminus, an arrangement consistent with Rem inside MMTV-infected cells, was degraded with somewhat lower efficiency. Additional constructs analyzed in mammalian cells confirmed that SP is degraded by the proteasome when a UbL region is attached to SP. However, SP itself does not appear to contain a proteasome degradation signal that can target a test protein (YFP-SP) to the proteasome (Fig. 6). Consistent with our other results, SP likely avoids proteasomal degradation following retrotranslocation because of failure to be recognized by E3 ligases and lack of ubiquitylation. On the other hand, uncleaved Rem behaves similarly in the presence and in the absence of the UbL, suggesting that Rem is polyubiquitylated and susceptible to proteasomal degradation.

SP functions without the activity of Derlin-1. Derlins are evolutionarily conserved proteins that span the ER membrane multiple times (8, 9). Although Derlins have been identified in multiple screens for ERAD components (12, 38, 58), Derlin-1 is not required for all ERAD substrates (54). Derlins have been proposed to be part of the retrotranslocon (8, 59), and yet their exact role is unknown. Recent evidence suggests that Derlins are related to rhomboid proteases, and yet they appear to have no proteolytic activity (60, 61). Other studies have implicated Derlins in the retrotranslocation of the polyomaviruses (23, 62, 63) and, in some experiments, the catalytic subunit of cholera toxin (53, 55). DN Derlin-1, but not DN Derlin-2, inhibits the function of both Rem and SP (Fig. 7), and yet CRISPR-Cas9-mediated knockout indicated that Derlin-1 was not required for Rem and SP function (Fig. 8). This result suggests that the effect of DN Derlin-1 on Rem activity is indirect. The DN Derlin-1 was constructed as a YFP fusion protein (53), which could sterically interfere with SP retrotranslocation by the p97 ATPase. DN Derlin-2 expression in Derlin-1-knockout cells had no significant effect on SP activity (Fig. S5), consistent with the conclusion that SP retrotranslocation is Derlin independent.

One of the best-studied retrotranslocation substrates that avoids proteasomal degradation is cholera toxin (64). AB_5_ first uses endocytosis to reach the Golgi complex and eventually the ER (22). In this process, the A subunit is cleaved into two components, cholera toxin A1 and -2 (CTA1 and -2) (65–67). Toxin binding to the binding immunoglobulin protein (BiP) chaperone in the ER leads to changes in CTA1 conformation through the cochaperone ERdj5 associated with the retrotranslocon (68). Release of CTA1 from BiP is then mediated by the nucleotide exchange factors Sil1 and Grp170, which allow toxin retrotranslocation into the cytosol (69). Although initial reports suggested that CTA1 requires Derlin-1 for retrotranslocation from the ER into the cytosol based on the DN Derlin-1 construct used here, this conclusion is being reconsidered (53, 55). Conformational changes in CTA1 may directly lead to its recognition by the E3 ligase Hrd1 and associated Sel1L protein to mediate retrotranslocation (70), perhaps using Hsp90 and its ATPase activity to promote ER membrane extraction and rapid folding at the cytosolic face of the ER membrane (71, 72). Rapid refolding has been proposed to prevent recognition of CTA1 by the proteasome (73). The involvement of Derlin-1 in CTA1 retrotranslocation is controversial, and it has been proposed that Derlin-1 makes substrate recognition more efficient (64). Further, the cytosolic p97 ATPase has been reported to provide the energy for membrane extraction of many ERAD substrates (2, 13), and yet this enzyme is dispensable for CTA1 dislocation to the cytosol (64, 65, 67). More recently, the chaperone Hsp90 has been shown to bind to CTA1 to facilitate its extraction from the ER membrane as well as rapid folding in the cytosol, at least in cell-free systems (71), but others dispute this claim (74). Membrane extraction of most ERAD substrates by the AAA ATPase p97 is followed by E3 ligase-mediated polyubiquitylation (5). Lack of detectable ubiquitylation for some substrates has been attributed to the absence of target lysines, and yet elimination of all CTA1 lysines had no effect on its retrotranslocation (75). In contrast, the deubiquitinase YOD1 has been reported to negatively regulate CTA1 membrane extraction (76). Thus, the exact means for CTA1 avoidance of ERAD remains unclear.

Use of ERAD by MMTV SP is unlike other viral signal peptides, which are subjected to intramembrane cleavage by SPP to facilitate release of viral structural proteins from the ER membrane (34–36). SP remains intact after p97 retrotranslocation to preserve the NES needed for its Rev-like function (26, 27). SP trafficking also differs from that of bacterial toxins and polyomaviruses. Unlike the bacterial toxins and SV40, which enter cells via retrograde transport (20, 21, 66, 77), Rem is synthesized on membrane-bound polysomes as a precursor protein that requires cleavage by signal peptidase to yield SP and a glycosylated C terminus (27, 32). Current results are consistent with the idea that some fraction of Rem is retrotranslocated, polyubiquitylated on the C terminus, and degraded by the proteasome by a more standard ERAD mechanism (Fig. 1B), and yet the majority of Rem is cleaved into SP and Rem-CT. The cleaved SP has a C-terminal membrane anchor (29), suggesting that the N terminus extends into the cytosol and directly binds to p97 (Fig. 1A) without the need for adapters, such as Derlins, Ufd1, or Npl4 (78). Such adapters normally would provide a link to E3 ligases, leading to polyubiquitylation and proteasomal degradation (Fig. 1B). This model is supported by (i) our failure to identify Derlins, transmembrane E3 ligases, or other common retrotranslocon components in a UBAIT screen; (ii) lack of evidence for involvement of Derlins using dominant negative or knockout approaches; (iii) inability to detect SP polyubiquitylation in immunoprecipitation experiments; and (iv) demonstration that addition of a ubiquitin-like domain leads to SP instability and sensitivity to proteasomal degradation. Thus, the p97-dependent, but Derlin-independent, mechanism used by MMTV-encoded SP is unique among pathogen-encoded proteins that evade ERAD.

## MATERIALS AND METHODS

### Cell lines and transfections.

Human embryonic kidney (293) cells (obtained from Glen Gaulton, University of Pennsylvania School of Medicine) were cultured at 37°C and 7.5% CO_2_ in Dulbecco’s modified Eagle’s medium (DMEM; Life Technologies, Inc.) supplemented with 10% fetal bovine serum (FBS), 100 U/ml penicillin, 100 U/ml streptomycin, 50 μg/ml gentamicin, and 2 mM l-glutamine. Cells were seeded in 6-well plates at 5 × 10^5^ cells/well for 24 h prior to transfection as previously described (27). The indicated amounts of DNA were transfected in triplicate (adjusted to a total of 6 μg DNA with the plasmid vector pcDNA3) by the calcium phosphate method, and cells were incubated for 48 h prior to preparation of lysates. In transfections using luciferase reporter vectors, 250 ng of pHM*Rluc* plasmid, which detects Rem activity, and 250 ng of the pGL3-control plasmid (Promega) were added in addition to various Rem expression constructs. The mouse mammary cell line HC11 (obtained from Jeff Rosen, Baylor College of Medicine) was selected to stably express an infectious MMTV provirus, pHYB-MTV (27), and grown in RPMI medium containing 10% FBS, 100 U/ml penicillin, 100 U/ml streptomycin, 50 μg/ml gentamicin, 2 mM l-glutamine, 0.5 μg/ml epidermal growth factor (Gibco), and 0.5 μg/ml insulin (Sigma). In some experiments, MG-132 (Boston Biochem) or lactacystin (Sigma) was added at a concentration of 10 μM at 36 h posttransfection for 12 h. All transfections were repeated at least twice with similar results.

### Production of SP UBAITs and mass spectrometry.

Plasmids for SP cloning as well as control plasmids were kindly provided by Jon Huibregtse (University of Texas at Austin) and used essentially as described previously (40). The SP coding region was obtained by PCR from the enhanced GFP (EGFP) vector containing GFP-SP (26, 27) using primers with flanking restriction sites (EcoRI and NotI for forward and reverse primers, respectively). The relevant plasmids and the SP insert were cut with EcoRI and NotI and ligated prior to transformation into competent *E. coli* cells. The TAP-SP-Ub and TAP-SP-UbΔGG constructs had three copies of the amino acid sequence for GGSG as a flexible linker between SP and the ubiquitin coding sequence. Plasmids were isolated from individual colonies and verified by sequencing, and large-scale purifications of plasmid DNA were performed prior to transfection. Eight 10-cm dishes were seeded with 1 × 10^7^ 293 cells and grown to confluence. Two 10-cm dishes were then used to individually transfect 3 μg of the SP constructs (TAP-SP-Ub, TAP-SP-UbΔGG, and TAP-SP) as well as the TAP-only control plasmid (adjusted to a total of 15 μg with pcDNA3 empty vector) by the calcium phosphate method. After 48 h, cells were harvested, resuspended in phosphate-buffered saline, pelleted, and then resuspended in 2 ml of 1% NP-40 lysis buffer containing a protease inhibitor cocktail (a 10-fold dilution of Sigma P2714 stock solution). Cells were lysed for 10 min on ice and subsequently subjected to centrifugation at 10,000 rpm for 10 min in a Beckman JA-20 rotor at 4°C to pellet cell debris. The supernatant was then incubated with 100 μl of an IgG slurry and left to bind overnight at 4°C. Bound beads were washed six times in 1% NP-40 lysis buffer containing protease inhibitor, resuspended in 2× concentrated SDS-containing loading buffer, and boiled for 10 min. Subsequently, the entire liquid fraction (50 μl) of the bead slurry was subjected to electrophoresis for a short time on a 12% SDS-containing polyacrylamide gel. The gel was stained with Coomassie brilliant blue overnight at 4°C, and all high-molecular-mass bands were excised and subjected to mass spectrometry at the UT Austin ICMB Proteomics Facility. The results were analyzed using Scaffold proteome software.

### CRISPR-Cas9-knockout cells.

To make a nickase version of Cas9 (56), a D10A mutation (79) was introduced by site-directed mutagenesis into the LentiCrispr ver. 2 (Addgene catalog no. 52961) vector. To make human *Derlin-1*-knockout cells, two guide RNAs (gRNAs) were designed to be specific for the *Derlin-1* coding sequence 5′ TCC CGG CGA TCA CGC GCT AT 3′ or for the complementary strand 5′-GTG GGT CGA AGA TGT CGG AC-3′ (selected using the algorithm at http://crispr.mit.edu/) and then inserted into the plasmid. Both gRNA-Cas9 D10A vectors (each 3 μg) were transfected into 293 cells. Single gRNA vectors were transfected to confirm that *DERL1* knockout was achieved only when the two gRNAs were cointroduced. After selection in 3 μg/ml puromycin and limiting-dilution cloning, single-cell colonies were analyzed using Western blotting to verify disruption of Derlin-1 expression.

### Reporter assays.

Reporter assays were performed as described previously (27, 32). Briefly, transfected cells were lysed in buffer provided in the Dual-Luciferase Reporter Assay System (Promega). Cells were subjected to three cycles of freezing and thawing, and cytosolic extracts were obtained after centrifugation. Protein concentrations were determined (Bio-Rad Protein Assay System), and ~40 μg of lysate was used to detect firefly and *Renilla* luciferase. Luciferase activities were determined using a Turner TD-20e luminometer (Turner Designs, Inc.) and normalized to 100 μg of protein. Values are reported as the average ± standard deviation from triplicate transfections after normalization of Rem-responsive *Renilla* luciferase activity for transfection efficiency as determined by the activity of the Rem-nonresponsive cotransfected pGL3-control vector expressing firefly luciferase.

### Western blotting and immunoprecipitation.

Whole-cell extracts of cultured mammary cells or pooled triplicate transfections were prepared by addition of 125 mM Tris-HCl, pH 6.8, 10% glycerol, 1% sodium dodecyl sulfate (SDS), 2.5% β-mercaptoethanol, 0.1% bromophenol blue and heating at 100°C for 5 min. Western blotting was performed essentially as previously described (27, 32). Total proteins (40 to 100 μg) were resolved in 10 to 12% polyacrylamide gels containing 1% SDS and transferred to nitrocellulose membranes. Membranes were preincubated with 20 mM Tris-HCl, pH 7.4, 137 mM NaCl (Tris-buffered saline [TBS]) and 0.1% Tween 20 (TBS-T) buffer containing 5% nonfat dry milk (TBS-TM) for 1 h and then incubated with primary antibody diluted in TBS-TM for 1 to 2 h followed by three washes with TBS-T for 10 min each. Horseradish peroxidase-conjugated secondary antibody in TBS-TM was added for 1 to 2 h, and then membranes were washed three times with TBS-T. Secondary antibody binding was detected using the Western Lightning Enhanced Chemiluminescent Reagent (PerkinElmer) as recommended by the manufacturer. For LI-COR detection, membranes were incubated with Odyssey Blocking Buffer (OBB) and TBS (1:2 ratio) followed by incubation with primary antibody diluted in OBB–TBS-T (1:2 ratio). The membranes were then washed three times with TBS-T and treated with fluorescently labeled IRDye secondary antibodies in OBB–TBS-T (ratio, 1:2) for 1 h followed by three washes in TBS-T and one TBS wash. Signals were detected using the Odyssey Imaging System according to the manufacturer’s instructions.

Antibodies were obtained from the sources indicated in parentheses: actin (Calbiochem or Sigma); GFP (Clontech); HA (Covance); Derlin-1, Derlin-2, and Derlin-3 (all from Sigma); goat anti-mouse IgG (Jackson ImmunoResearch, Inc.); and goat anti-rabbit IgG (Abcam, Inc., or Jackson ImmunoResearch). IRDye 800CW goat anti-mouse IgG and IRDye 680RD goat anti-rabbit IgG were from LI-COR. The MMTV SP-specific antibody was prepared in rabbits as previously described (27).

For immunoprecipitation experiments, GFP-Rem and HA-ubiquitin (HA-Ub) expression constructs (3 μg each) were transfected into 1 × 10^6^ 293 cells. After 48 h, two-thirds of the cells were lysed in 250 μl of NP-40 lysis buffer (0.1 M Tris-HCl, pH 7.5, 0.1 M NaCl, 1% NP-40, and 1 mM dithiothreitol [DTT]) for 20 min on ice. The remaining cells were used for Western blotting. T25N50 buffer (25 mM Tris-HCl, pH 7.5, and 50 mM NaCl) was added to 90% of the lysed supernatant to a final volume of 300 μl. The lysate was incubated with prebound GFP antibody-agarose beads (A/G) (Santa Cruz Biotechnology) for 2 h at 4°C. After washing three times in NP-40 lysis buffer, SDS loading buffer was added to immunoprecipitates and cell lysates. Samples were boiled for 5 min prior to centrifugation and analyzed by SDS-containing polyacrylamide gel electrophoresis prior to Western blotting.

### Plasmid constructs.

The N-terminally GFP-tagged (GFP-Rem or GFP-SP) and untagged Rem expression plasmids have been reported previously (28). The reporter plasmids pHM*Rluc* (26) and pGL3-control (Promega) have been described for transient-transfection assays (26, 28). The p97 DN (p97QQ) (41) expression vector was kindly provided by Yihong Ye (National Institutes of Health). The C-terminally GFP-tagged α-1 antitrypsin mutant (NHK-GFP) or the mutant lacking glycosylation sites (NHKQQQ-GFP) (80) was used as a control for its activity with Derlin-2 or p97QQ, respectively; these plasmids were provided by N. Hosokawa (Kyoto University). The plasmid encoding fast-folding YFP was obtained from B. S. Glick (University of Chicago). The coding sequences were cloned into the plasmid pGEM-3Zf(+) (Promega) for expression *in vitro*. The CEN plasmid YCplac33 was used for the budding yeast experiments, whereas the mammalian expression vector pcDNA5 (Life Technologies, Inc.) was used for experiments in cultured cells. In the model proteins, UbL domains from *Saccharomyces cerevisiae* Rad23 or its human homolog hHR23B were connected to the N or C terminus of *E. coli* dihydrofolate reductase (DHFR) or the N terminus of YFP through the linker sequence (VDGGSGGGS). Tails were attached to the N or C terminus of DHFR or the C terminus of YFP through a 2-amino-acid linker (PR). The tails were derived from Rem, SP, or the first 29 or 89 amino acids of *S. cerevisiae* cytochrome *b*_2_ (designated 35 and 95, respectively, after addition of 6 histidine residues). The coding sequences of the model proteins were cloned into the plasmid pGEM-3Zf(+) (Promega) for expression *in vitro* or into the vector pcDNA5 (Life Technologies, Inc.) for transfections into HEK 293 cells. The vector pcDNA5 contained the CMV promoter upstream of the coding regions for mCherry and YFP separated by an internal ribosomal entry site (IRES) derived from the encephalomyocarditis virus (81).

### Protein expression, purification, and degradation assay.

Protein substrates were derived from *E. coli* DHFR, *S. cerevisiae* cytochrome *b*_2_, Rad23, and MMTV Rem. Yeast proteasomes were purified from *S. cerevisiae* strain YYS40 (*MAT***a*** rpn11*::*RPN11* 3×FLAG-*HIS3 leu2 his3 ura3 trp1 ade2 can1 ssd1*) by immunoaffinity chromatography using FLAG-specific antibodies (M2 agarose affinity beads; Sigma) as described previously with modifications (82). For *in vitro* degradation experiments, radioactive substrates were expressed from a T7 promoter using a coupled *in vitro* transcription-translation reaction (TNT Coupled Reticulocyte Lysate System; Promega) containing [^35^S]methionine according to the manufacturer’s protocol. After synthesis, the substrates were partially purified by high-speed centrifugation followed by precipitation in 2 volumes of saturated (NH_4_)_2_SO_4_ as described previously (49). Degradation assays with radiolabeled substrates were performed as described earlier (49). Briefly, the assay mixtures at 30°C contained radiolabeled model proteins together with 40 nM purified yeast proteasomes in a reaction buffer with a creatine phosphate and creatine kinase ATP-regenerating system. Samples were removed at designated times, added to SDS-containing sample buffer to stop the reaction, and analyzed by SDS-polyacrylamide gel electrophoresis. Protein amounts were determined by electronic autoradiography (Instant Imager; Packard) and normalized to the initial input. The degradation curves were nonlinearly fitted to a single exponential decay using the software package Kaleidagraph (version 4.1; Synergy Software).

### Statistical analysis.

All experiments were repeated at least twice with similar results. Pairwise comparisons of reporter assays were analyzed by a two-tailed Student *t* test. Values of <0.05 were considered significant and have been indicated by an asterisk in the figures. Nonsignificant values have been designated NS.
