# Supplementary figures and images for: Mouse Mammary Tumor Virus Signal Peptide Uses a Novel p97-Dependent and Derlin-Independent Retrotranslocation Mechanism To Escape Proteasomal Degradation
Source: mBio. 2017 Mar 28;8(2):e00328-17. doi: 10.1128/mBio.00328-17 (PMC5371415; doi:10.1128/mBio.00328-17)

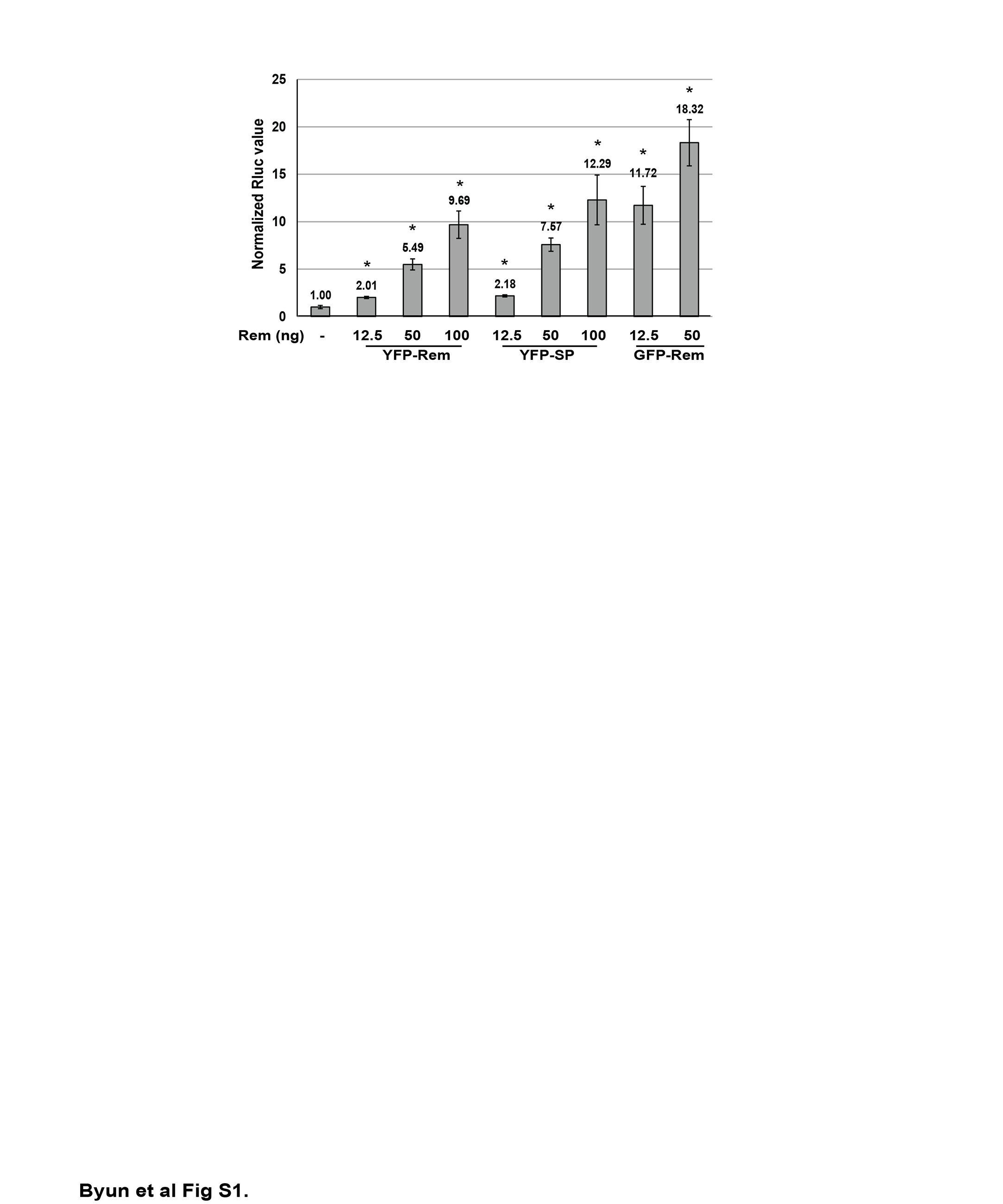

Supplement: FIG S1 [file mbo002173257sf1.tif]

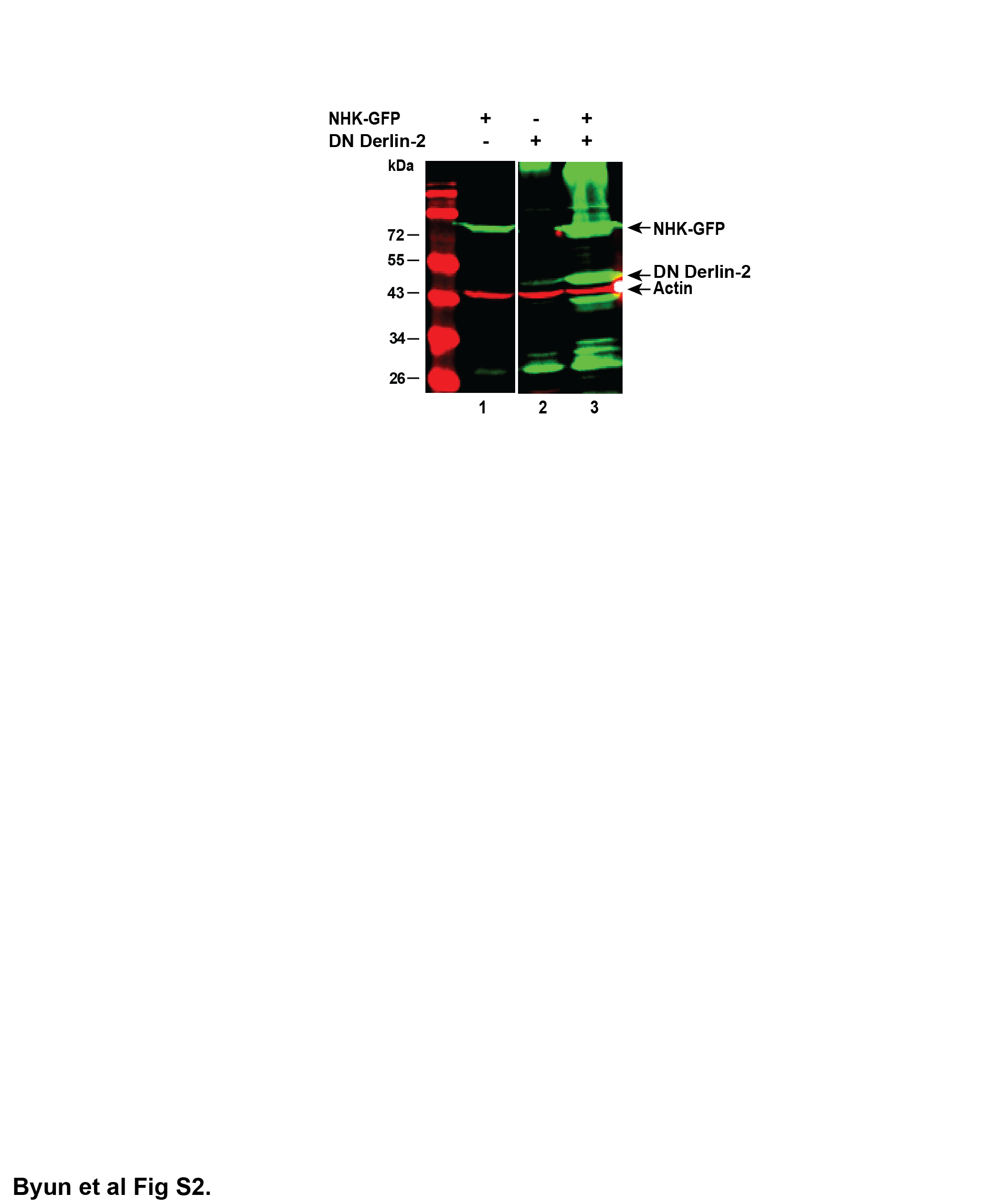

Supplement: FIG S2 [file mbo002173257sf2.tif]

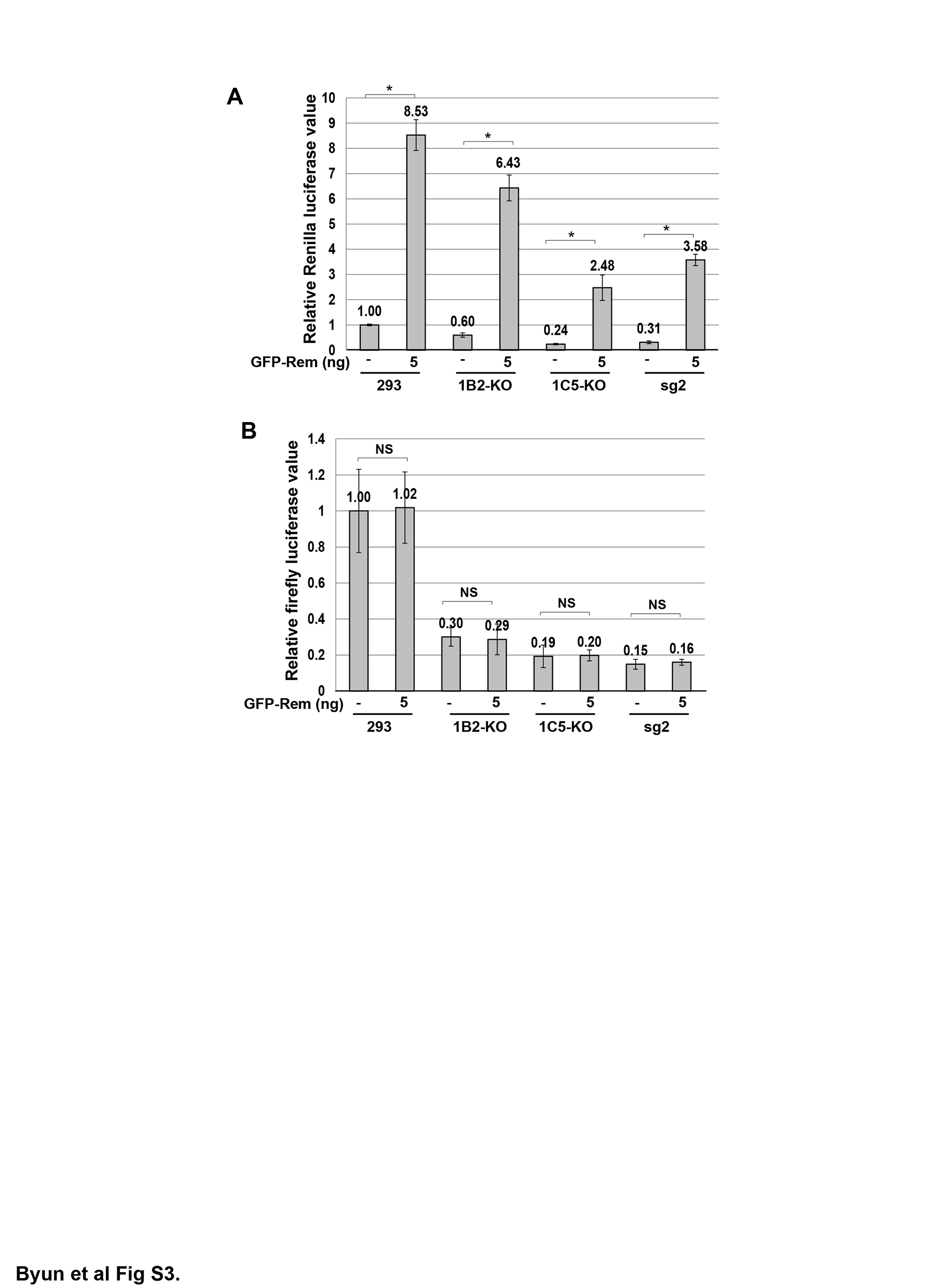

Supplement: FIG S3 [file mbo002173257sf3.tif]

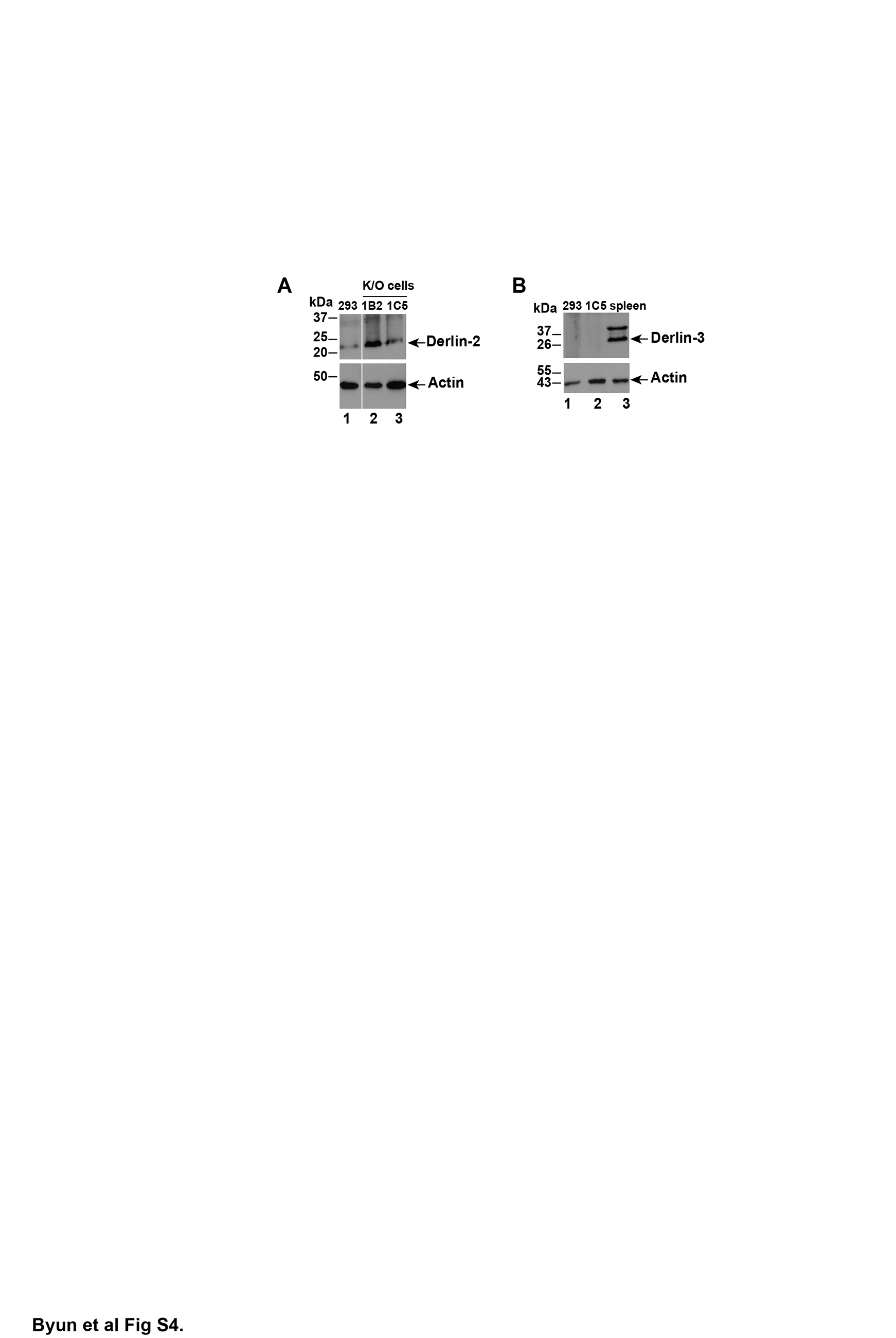

Supplement: FIG S4 [file mbo002173257sf4.tif]

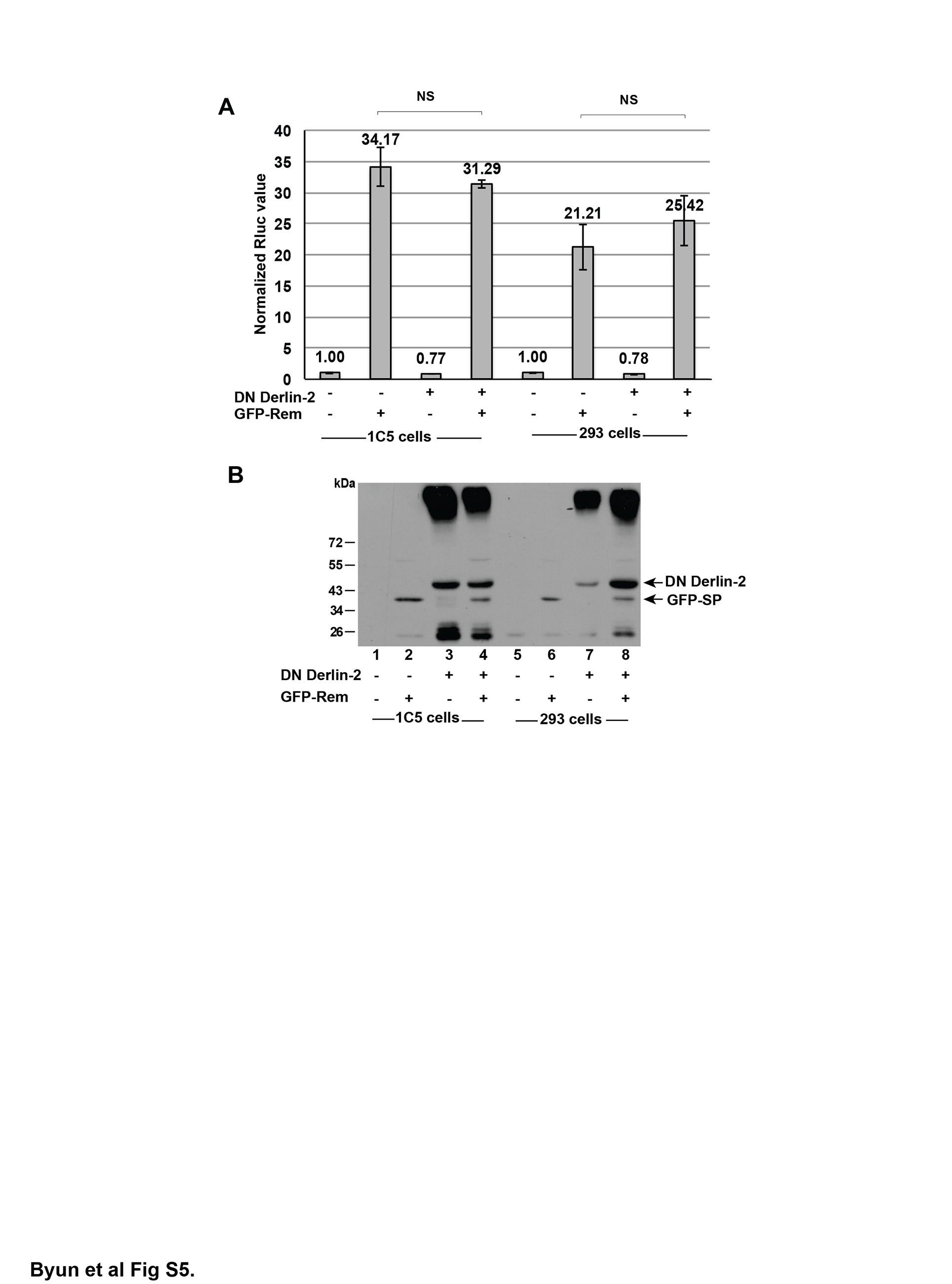

Supplement: FIG S5 [file mbo002173257sf5.tif]
